# Context matters: how river typology shapes biotic responses to fine sediment pressure

**DOI:** 10.1007/s10980-026-02297-z

**Published:** 2026-01-28

**Authors:** Kate L. Mathers, Morwenna McKenzie, Adrian L. Collins, Jessica M. Durkota, J. Iwan Jones, John F. Murphy

**Affiliations:** 1 Geography and Environment, Loughborough University, Loughborough, UK; 2 Rothamsted Research, North Wyke, Okehampton, Devon UK; 3 Environment Agency, Teville Gate House, Worthing, BN11 1UR UK; 4 School of Biological and Behavioural Sciences, Queen Mary University of London, London, E1 4NS UK

**Keywords:** Context-specific, Ecological threshold, Environmental filtering, Macroinvertebrate communities, Plasticity, Sediment

## Abstract

**Context:**

Excess fine sediment is a global stressor affecting freshwater biodiversity. However, little consideration has been given to how large-scale landscape controls and temporal variability may influence the effect of fine sediment deposition and storage on biological communities.

**Objectives:**

We assess if ecological responses to deposited fine sediment are spatially and temporally consistent through the application of the river typology approach.

**Methods:**

We used 2,940 records from 391 wadable streams across England and Wales to identify taxonomic and functional community composition change points, in addition to individual family responses along the fine sediment gradient. We also examined the association of taxonomic and functional community diversity metrics and biomonitoring metrics with deposited fine sediment coverage.

**Results:**

Mid-altitude rivers displayed a lower community threshold (~ < 10% fine sediment cover) of deposited fine sediment before the majority of community change occurred, whilst lowland rivers were more tolerant (20–25%). Critically, we found that both mid-altitude river types demonstrated no association with two fine sediment stressor-specific metrics and that some community metrics displayed a positive association with increasing fine sediment cover.

**Conclusions:**

Community and family level responses to deposited fine sediment are non-linear, which can be characterized effectively by river typologies and most notably altitude groupings. Low levels of deposited fine sediment may not act as a stressor in mid-altitude catchments as these may be resource limited. Our research underlines the need to consider context-specific effects of fine-grained sedimentation rather than seeking to generalise stressor effects.

**Supplementary Information:**

The online version contains supplementary material available at 10.1007/s10980-026-02297-z.

## Introduction

Globally, freshwaters are under increasing pressures, with biodiversity declines far outstripping their terrestrial and marine counterparts (Reid et al. 2019; Albert et al. 2021). One potential cause for this decline is excess fine sediment (particles < 2 mm: Mckenzie et al. 2024). Although a natural facet of ecosystem functioning, contemporary levels of fine sediment storage now far exceed background levels due to changes in river flow regimes and land use conversion (Walling and Fang 2003; Foster et al. 2011). Inputs of fine sediment are anticipated to be further exacerbated in the future due to changes to rainfall and runoff regimes associated with climatic change (Burt et al. 2016; Li et al. 2020). As such, managing the ecological effects of excess fine sediment in lotic systems is a global challenge that researchers and environmental regulators need to address in order to conserve freshwater biodiversity and the ecosystem services provided (Haase et al. 2023; Lynch et al. 2023).

The source of fine sediment and therefore the composition (organic / inorganic) influences its potential ecological implications, with alterations to the physiochemical conditions of the streambed including nutrients, oxygen concentrations and resource availability being possible (Greig et al. 2005; Von Bertrab et al. 2013; dos Reis Oliveria et al. 2019; Mckenzie et al. 2025). Excess fine sediment deposition also leads to the homogenisation of instream habitats, infilling of interstitial pore space and limited hydraulic connectivity (Wood and Armitage 1997). Fine sediment is widely acknowledged to affect all trophic levels (Mustonen et al. 2016) from diatoms (Jones et al. 2014) and macrophytes (Jones et al. 2012b), through to invertebrates (Jones et al. 2012a) and fish (Kemp et al. 2011). Although the entire food web is affected, invertebrates are widely used as bioindicators, acting as a proxy for wider ecosystem condition and habitat quality. A number of taxonomic measures (e.g. Ephemeroptera, Plecoptera and Trichoptera based indices, taxa richness; Doretto et al. 2018) have been proposed as indicators for fine sediment stress in addition to individual bespoke sediment-specific indices which have been developed to diagnose whether fine sediment stress is acting on a river site (e.g. Relyea et al. 2012; Extence et al. 2013; Doretto et al. 2018; Gieswein et al. 2019). Functional trait measures have also been advocated as a complementary measure to traditional taxonomic approaches because they provide insights into the mechanisms causing community change, rather than simply observing that a change has occurred (Culp et al. 2011; Murphy et al. 2017; Paz et al. 2023). Often the utility of these metrics is demonstrated on either a geographically restricted test dataset (Glendell et al. 2014; Mckenzie et al. 2022) or on a larger more extensive test dataset without any direct consideration / evaluation of the variation in the performance of the metric across natural environmental gradients (Murphy et al. 2015). A more considered and comprehensive assessment of their applicability to different types of river has not yet been tested.

Despite the widely acknowledged negative effects of excess deposited fine sediment, currently there are few standards or guidelines globally for assessing thresholds over which ecological degradation occurs (in marked contrast to suspended sediment or flow discharges; Mondon et al. 2021). Moreover, concerns have been raised that stressor-responses of aggregated community metrics, may obscure species-specific responses which may include different magnitudes, direction of response and unaffected taxa, (King and Baker 2010, 2011). As such, utilising threshold analyses as a complementary tool to biomonitoring efforts enables the specific taxa that are positively and negatively responding to the stressor gradient to be identified alongside community change points (King and Baker 2010; Wagenhoff et al. 2017; DeVilbiss et al. 2025). Ecological threshold analysis could therefore represent a valuable tool to aid monitoring and management protocols for fine sediment and landscape ecology, particularly through the creation of safe operating spaces (Groffman et al. 2006).

A holistic approach, using a data derived river typology, is increasingly being advocated for in the assessment of environmental factors driving ecological community change. The river typology approach categorises rivers based broadly on their size, underlying geology, and catchment altitude (Solheim et al. 2019). This method has been recently adopted to assess abundance trends of specific taxa (Powell et al. 2023), nutrient thresholds (Poikane et al. 2019) and multiple anthropogenic stressors (Lemm et al. 2021). It is anticipated that the approach should be readily transferable for assessing the ecological implications of fine sediment at the landscape scale, but this has yet to be tested. Geological conditions are likely to control the delivery and infiltration of fines into the riverbed (Hunter et al. 2024). For example, calcareous rivers are often groundwater fed, and prone to fine sediment saturation due to a lack of flushing flows associated with buffered flow regimes (Monden et al., 2021; 2024). Conversely, siliceous rivers are often surface water fed and thus more susceptible to fluctuating flows with extreme low flows during drought periods (Berrie 1992).

Elevation will also exert specific controls on the way ecological communities respond to environmental pressures. In general, upland areas are often dominated by high gradient, high energy systems characterised by coarse grained riverbeds and support communities more susceptible to environmental change including fine sediment pressure (Larsen et al. 2009; Mathers et al. 2022). In contrast, lowland areas are typically characterized by greater numbers of fine-grained riverbeds associated with lower slopes and therefore lower energy systems that facilitate the infiltration of fine sediment. In addition, lowland systems are typically subjected to greater anthropogenic influences including intensive agricultural land use, nutrient enrichment and river channel modification. Ecological communities inhabiting lowland rivers may therefore have undergone previous environmental filtering as a result of long-term inputs of high amounts of fine sediment (Matthaei et al. 2006; McKenzie et al. 2024).

The river typology approach represents a useful management and monitoring tool in landscape ecology given that rivers can be readily categorised and therefore ecological responses can be assessed within the typology framework reducing the environmental variation associated with broad spatial controls (Jupke et al. 2022; Powell et al. 2022). Evidence increasingly suggests that the effects of fine sediment cannot be generalised (Matthaei et al. 2006; Larsen et al. 2009; Mathers et al. 2022; 2024a, b; Mckenzie et al. 2024) and that context-specific effects are more likely (Nguyen et al. 2023; Snåre et al. 2024). Similarly, sampling season is expected to reflect both biological and physical influences associated with taxon life-histories (Johnson et al. 2012), flow regimes (Buendia et al. 2014), instream sediment storage (Cotton et al. 2006) and intra-annual variations in fine sediment inputs (Davis et al. 2024). Recent research by Mathers et al. (2023) in temperate streams has shown that resource partitioning and richness (taxonomic, functional and EPT) supported by fine sediment habitats varies seasonally, with the greatest richness occurring during autumn months. Therefore community level sensitivity to fine sediment inputs is likely to demonstrate some seasonality, but this is not always accounted for directly within biomonitoring programmes (with seasonal samples often being pooled) when assessing the ecological health of rivers (Carlson et al. 2013).

In this, first large-scale study, we consider the role of context specificity in defining fine sedimentation responses associated with broad scale environmental factors. Specifically, we sought to examine: 1) how taxonomic and functional communities vary in response to deposited fine sediment across different river types and sampling season; 2) if individual taxa have consistent responses to deposited fine sediment across different river types and sampling season, and; 3) the association of invertebrate community indices with deposited fine sediment across different river types and sampling seasons.

## Methods

### Datasets

**Fig. 1:**
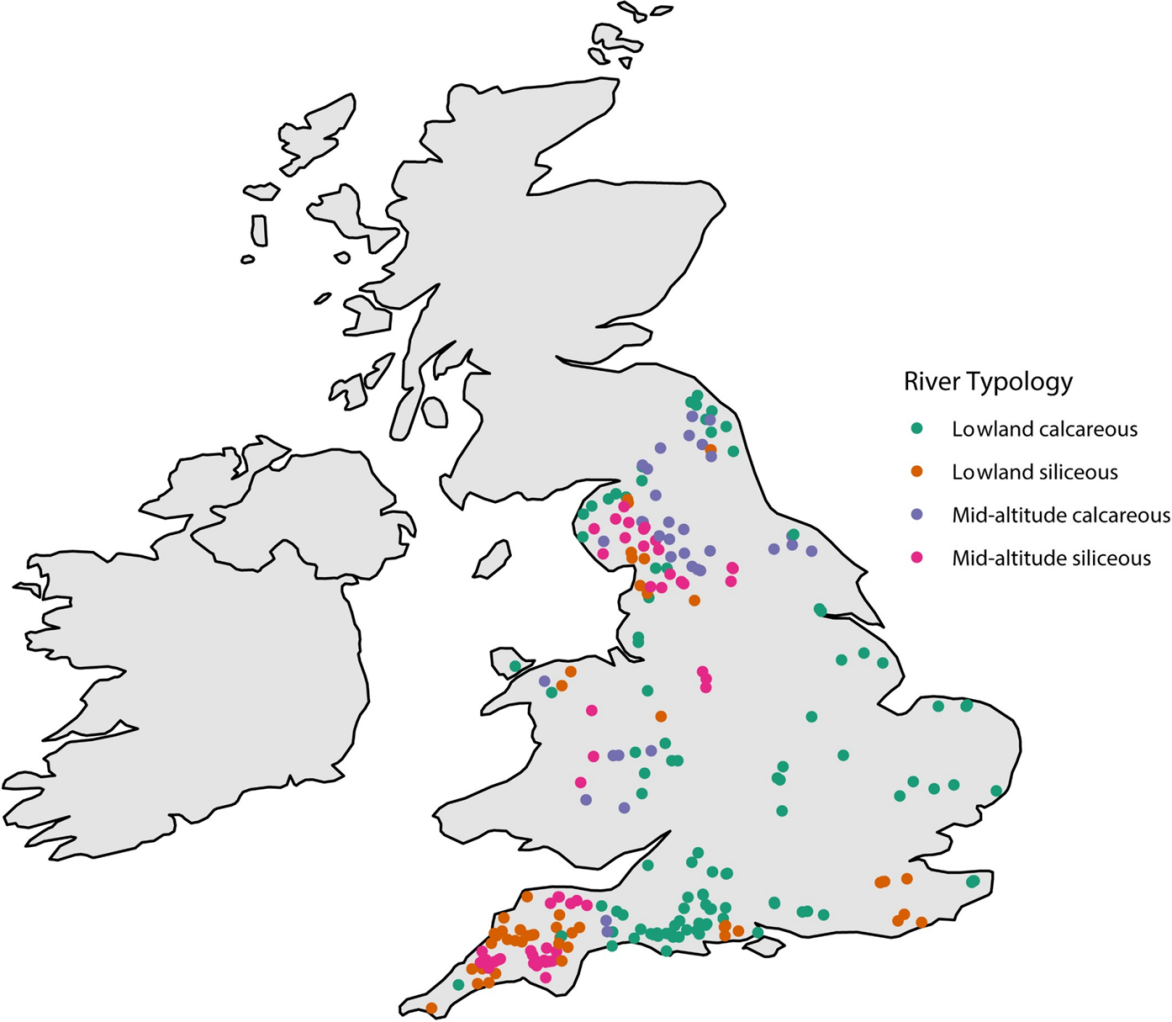
Map of the sample locations (*n* = 392) in England and Wales coloured by river typology. Number of sites within each typology is as follows: lowland calcareous = 168, lowland siliceous = 85, mid-altitude calcareous = 56 and mid-altitude siliceous = 83

Data from streams was collated from two sources; the Environment Agency of England ecological monitoring database, and academic research (Murphy et al. 2015, 2017; Jones et al. 2017). Data comprised paired biological (invertebrates), and environmental (visual fine sediment cover %) data collected during the same sampling occasion at 391 sites across England and Wales (Fig. 1). This consisted of a total of 2,940 samples. Data were collected either in spring (March – May) or Autumn (September – November) with no seasonal bias present in the dataset (*n* = 1491 in spring and *n* = 1438 in autumn). We restricted the Environment Agency dataset to 2002 – 2019 as, in 2002 the Environment Agency switched from recording abundance on a log_10_ ordinal scale to exact abundances (sensu Powell et al. 2023).

In all data sources, reach scale substrate composition was characterised using a visual estimate of percentage cover of the bed surface across a range of size classes (e.g., boulder, cobble, gravel etc.), with the percentage of fine sediment calculated by aggregating all substrate categories < 2 mm in diameter (clay, silt, sand). Obtaining reference conditions of fine sediment is difficult and in many instances benchmark datasets do not exist. Moreover, fine sediment levels are highly dynamic, reflecting episodic fine sediment transport and deposition events (Foster et al. 2011; Davis et al. 2022). As such, here and in the majority of studies, it is difficult to disentangle natural levels from excess loadings, which has led to the increasing application and development of biological indices that aim to track further, or elucidate ecological change associated with fine sediment pressures across the globe (e.g. Doretto et al. 2018; Gieswein et al. 2019). It should be noted, however, that the majority of streams have contemporary fine sediment inputs that far exceed background levels (Foster et al. 2011).

Invertebrates were collected using standard 3-min kick samples (1 mm mesh size), preserved, and returned to the laboratory for identification and enumeration following Environment Agency protocols (Environment Agency 2014). Data were converted to relative abundance (Chen and Olden 2020) and resolved to family level to account for the mixed levels of identification (Everall et al. 2017; Stubbington et al. 2022). Sample locations were filtered (either prior to acquisition or during data collection) to ensure a reduction in co-occurring stressors which may confound or interact with the effects of deposited fine sediment. Detailed descriptions of sampling methods and data filtering for both data sources can be found in Supplementary 1.

Macroinvertebrate functional trait data were compiled for each recorded family from Tachet et al. (2010). Tachet et al. (2010) describes the affinity of each taxa for different trait modalities within each of 11 traits using a fuzzy coded scoring system (Table S1). Affinities of all recorded genera were averaged to provide a family score (sensu Gayraud et al. 2003). Twenty-two (of 150) taxa were unable to be assigned scores, which accounted for only 0.06% of the total abundance. Trait modality scores were standardised following ‘fuzzy coding’ standardisation (Chevene et al. 1994) using the ‘*prep.fuzzy’* function in the ade4 R package (Thioulouse et al. 2018), such that the sum of trait modality affinity scores within each trait for a given taxon summed to 1. Subsequently the traits x abundance matrixes were combined to calculate a community weighted means (CWM) of all trait modalities using the ‘dbFD’ function in the FD R package (Laliberté et al. 2014).

### River typology

Sites were categorised into typology groups using data held by the Environment Agency and criteria from the EU Water Framework Directive’s river typologies descriptions (Water Framework Directive UKTAG 2003) which includes the dominant catchment geology and mean catchment altitude (see Solheim et al. 2019 for more details). The classification was designed to ensure cross country comparisons of a reduced number of river types using a broad set of variables that could capture the inherent natural variability of river systems. This board typology was developed using dialogues and data provision from all European Union countries to ensure a generic typology that encompassed 80% of rivers. Sites dominated by chalk or limestone were classified as ‘Calcareous’, and those by clay or hard rock as ‘Siliceous’. Sites with a mean catchment altitude of ≥ 200 m were categorised as ‘mid-altitude’ and those < 200 m as ‘lowland’. There were no samples from the third altitude group of highland (> 800 m). This provided four typologies for our analyses: 1) Lowland Calcareous; 2) Lowland Siliceous; 3) Mid-altitude Calcareous, and; 4) Mid-altitude Siliceous (Fig. 1). Note we excluded the third geology of organic (dominated by peat) from our analysis as this accounted for only 36 samples. The typology of river size was not included in the analysis as we focused on wadable rivers sampled by kick sampling (as standard protocol for statutory monitoring purposes) thereby limiting the influence of size within our dataset. There were no large rivers (> 1,000 km^2^) and 85% of samples were classified as small (< 100 km^2^). Sample breakdown by season and typology is presented in Table 1.

**Table 1:** Summary of sample breakdown used in study

Season	Lowland calcareous	Lowland siliceous	Mid-altitude calcareous	Mid-altitude siliceous
All	1375	486	320	748
Spring	698	255	156	382
Autumn	677	231	164	366

### Statistical analysis

Each analysis was performed separately for the four river types to quantify the consistency of invertebrate responses (for comparable analysis based on the entire dataset please see McKenzie et al. 2024). Threshold Indicator Taxa Analysis (TITAN: Baker and King 2013) was carried out using the *TITAN2* package (Baker et al. 2015). TITAN is a nonparametric method which uses a resampling technique to detect abrupt change points of relative abundance and occurrence across an environmental gradient (King and Baker 2010; Baker and King 2013). TITAN analysis is relatively robust to uneven sampling across an environmental gradient, but the performance can be affected by extreme imbalances i.e. where parts of the gradient are underrepresented in the data (Baker and King 2014). Whilst TITAN incorporates the use of bootstrapping to reduce the influence of outliers, results can still be sensitive to zero-inflated or extreme skewed abundance distributions, which may affect the detection of pure and reliable indicator taxa. Function parameters were set as 250 random permutations (*numPerm*) and 500 bootstrap replicates (*nBoot*) (Porter-Goff et al. 2013; Khamis et al. 2014; Lencioni 2018). A taxon or trait was identified as either responding positively (*z* +) or negatively (*z*-) to the deposited fine sediment gradient if: a) the change in frequency and relative abundance was the same for ≥ 95% of all bootstrap samples (i.e. pure), and; b) ≥ 95% of all bootstrap samples were significantly different from a random distribution (*p* < 0.05) (i.e. reliable). The sum of all IndVal *z* scores (sum*z*) was used as an indicator of taxonomic or functional community level ‘change point’ by identifying peaks along the gradient associated with the maximum decline or increase in frequency and/or relative abundance of negative (sum*z*-) and positive (sumz +) responders, respectively (King et al. 2016; Monk et al. 2018). Only community trait responses were examined (no individual trait responses were assessed unlike taxa) as selection pressures do not act on single traits, but on organisms possessing many interacting traits (Verberk et al. 2013) which is reflected in the equivocal individual trait-sediment relationships reported in the literature (see Murphy et al. 2017; Wilkes et al. 2017 for summaries).

Twelve taxonomic, functional and biomonitoring metrics were calculated to determine the consistency of responses in commonly employed biodiversity metrics to fine sediment. The taxonomic indices calculated were taxa richness, Ephemeroptera, Plecoptera and Trichoptera (EPT) richness, and %EPT. In addition, two sediment-specific biomonitoring metrics were calculated; Proportion of sediment-sensitive invertebrates – PSI (Extence et al. 2013) and the Empirically-weighted PSI—E-PSI (Turley et al. 2016). Given PSI has been shown to have strong correlations with flow (Lotic Index for Flow Evaluation—LIFE; Extence et al. 1999) and water quality measures (Walley Hawkes Paisley Trigg -WHPT; Walley and Hawkes 1996) when analysed over large scales at many sites (Murphy et al. 2015; Turley et al. 2015, 2016; McKenzie et al. 2022), these measures were also calculated. Two WHPT metrics were calculated; WHPT-Total (abundance-based adaptation of the globally utilised BMWP index) which is derived from the sensitivity of invertebrate families to organic pollution but can be affected by other factors; and the WHPT Average Score Per-Taxon (WHPT-Total divided by the number of scoring taxa, coded WHPT-ASPT; Paisley et al. 2014) which principally reflects organic pollution. Five functional diversity indices were calculated using the *FD* package comprising; functional richness (FRic; the minimum convex hull encompassing all species), functional dispersion (FDis; representing the mean distance in multidimensional trait space of individual species to the centroid of all species), functional evenness (FEve; reflecting the regularity in which species are distributed across functional space), functional divergence (FDiv; representing how abundance is distributed within the volume of functional space occupied by species) and Rao’s quadratic entropy (RaoQ; reflecting the mean distance among species weighted by species abundance; Villéger et al. 2008; Laliberté and Legendre 2010).

Spearman’s rank correlation (due to non-normal data distributions) was applied to assess the association of the indices against visual deposited fine sediment %. Pairwise correlations were corrected for multiple comparisons using the Holm-Bonferroni correction (Holm 1979). All analyses were conducted in the R environment (R Development Core Team, 2022). Both TITAN and correlation analysis were performed on all data (spring and autumn combined) and, individually by season, to assess if invertebrate responses were consistent.

## Results

### Community and taxa responses to deposited fine sediment by river typology and season

**Fig. 2:**
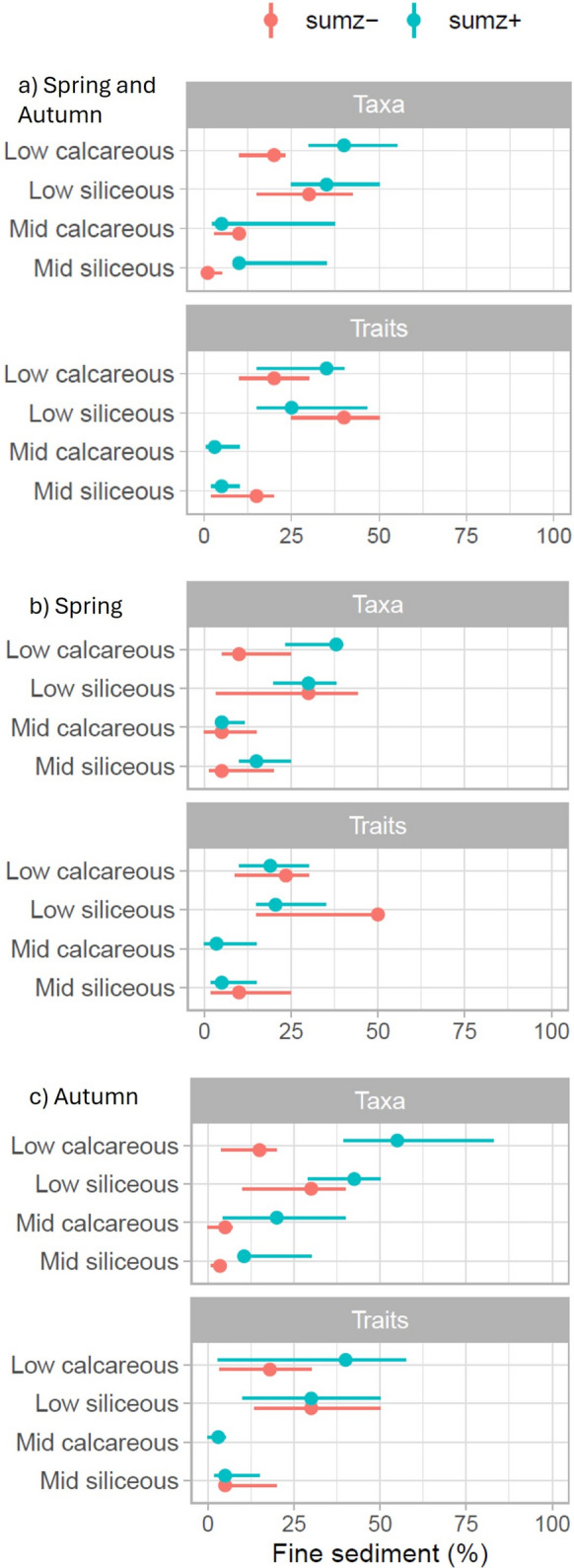
Observed sumz- (red) and sumz + (blue) maxima (i.e., change points) identified by Threshold Indicator Taxa Analysis (TITAN) for taxonomic (taxa) and functional (traits) measures of invertebrate communities in; **a** spring and autumn combined, **b** spring and, **c** autumn along the deposited fine sediment gradient (% visual cover). Where present, peak change points indicated as circles with 5th and 95th percentile distributions as horizontal lines. Change points are filtered to include only pure and reliable taxa / traits. No community responses for Z- traits were recorded for seasonal mid-calcareous data.

Taxonomic and functional measures demonstrated contrasting responses along the deposited fine sediment gradient associated with river typology (Fig. 2a). Lowland sites displayed less sensitivity, demonstrated by higher community change points, than in mid-altitude rivers. Change points for positively responding (sum*z* +) taxa generally demonstrated larger confidence intervals particularly for those in mid-altitude rivers. In most instances, negatively responding (sum*z*-) taxa demonstrated a lower change point than positively responding taxa, whilst the converse was true for traits, with positively responding traits displaying a lower change point. Overall, change occurred after 20% deposited fine sediment coverage for lowland rivers, whilst most change had occurred at around 10% in mid-altitude rivers (Fig. 2a).

**Fig. 3:**
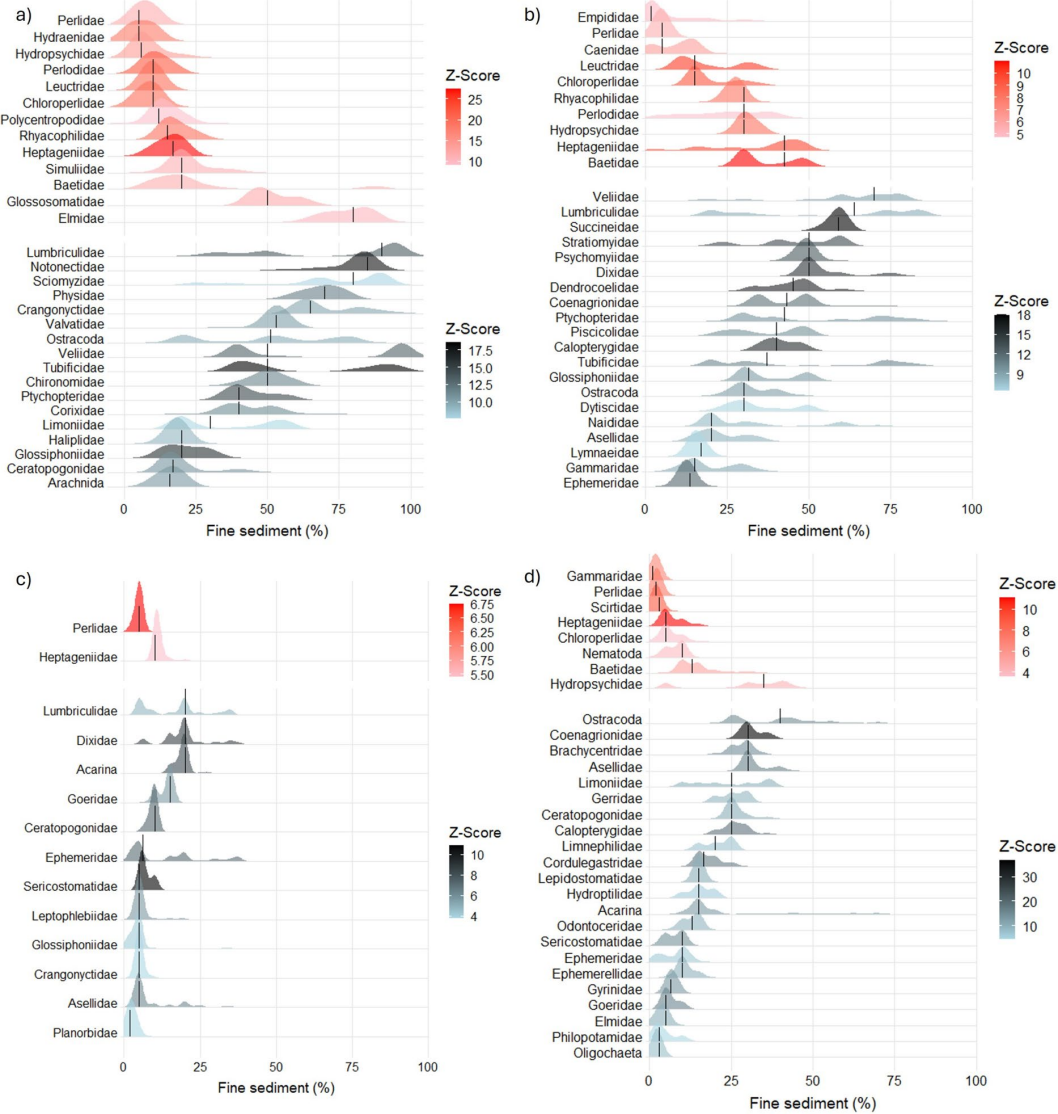
Individual taxa response plots from Threshold Indicator Taxa ANalysis (TITAN2) along the deposited fine sediment gradient (% visual cover) for spring and autumn data combined for; **a** lowland calcareous; **b** lowland silicious, **c** mid-altitude calcareous and, **d** mid-altitude silicious. Taxa that responded negatively to the gradient are shown in red, while positive indicator taxa are shown with blue. Taxa change points (across 999 bootstrapped replicates) are visualized as a probability density function with colour intensity scaled according to the magnitude of the response (i. e., its standardized z-score). Comparable Spring and Autumn only plots can be found in Supplementary material (Figures S1 and S2). Maximum number of taxa visualised was limited to thirty.

Considering the individual taxa driving the change points, taxa identified as negatively responding indicators of deposited fine sediment were dominated by those from the EPT orders, whilst positively responding taxa comprised predominately Diptera (Fig. 3). Several taxa were found to consistently respond negatively in all river types; notably Perlidae and Heptageniidae. Perlidae demonstrated a similar highly sensitive change point in all rivers (ca. 5%), whilst Heptageniidae was more variable demonstrating a low % fine sediment change point in mid-altitude rivers (Fig. 3c) and a higher % fine sediment change points in lowland rivers (Fig. 3a, b). Other notable taxa included Hydropsychidae and Chloroperlidae which were indicators (z-) in three river types (not mid-altitude calcareous) and Rhyacophilidae for lowland rivers (Fig. 3). Interestingly, Gammaridae displayed contrasting responses in siliceous rivers, being a highly sensitive negative responder in mid-altitude rivers (Fig. 3d) but responded positively in lowland rivers (Fig. 3b). There were considerably more taxa identified as positively responding (i.e. tolerant) than negatively responding (i.e. sensitive) across all river types. Acarina, Asellidae, Ephemeridae, and Lumbriculidae were positively responding indicators of deposited fine sediment in all rivers. A further nine taxa were identified as positively responding in three out of the four river types.

When sampling season was considered, clear differences were evident for some change points (Fig. 2b, c). In general, negatively responding taxonomic communities (sum*z*-) demonstrated fairly consistent responses regardless of the season for all river types. In contrast, positively responding taxonomic communities displayed lower change points in spring for three river types (not mid-altitude siliceous), which was most evident for lowland calcareous. Functional community responses were more consistent across the seasons for mid altitude rivers with low change points for both positive and negative responders (Fig. 3b, c). In lowland rivers, change points of negativly responding traits were higher than positively responding traits in spring, whereas the opposite was true in autumn (except for siliceous rivers where the change points were approximately equal between positively and negatively responding traits).

Differences in the indicator taxa by season were evident with some occurring in one season but not the other (Figure S1, S2). A number of taxa occurred in both seasons, some with similar thresholds whilst others demonstrated variable change points between seasons (Figure S1, S2).

### Ecological indicators of deposited fine sediment by river typology and season

**Fig. 4:**
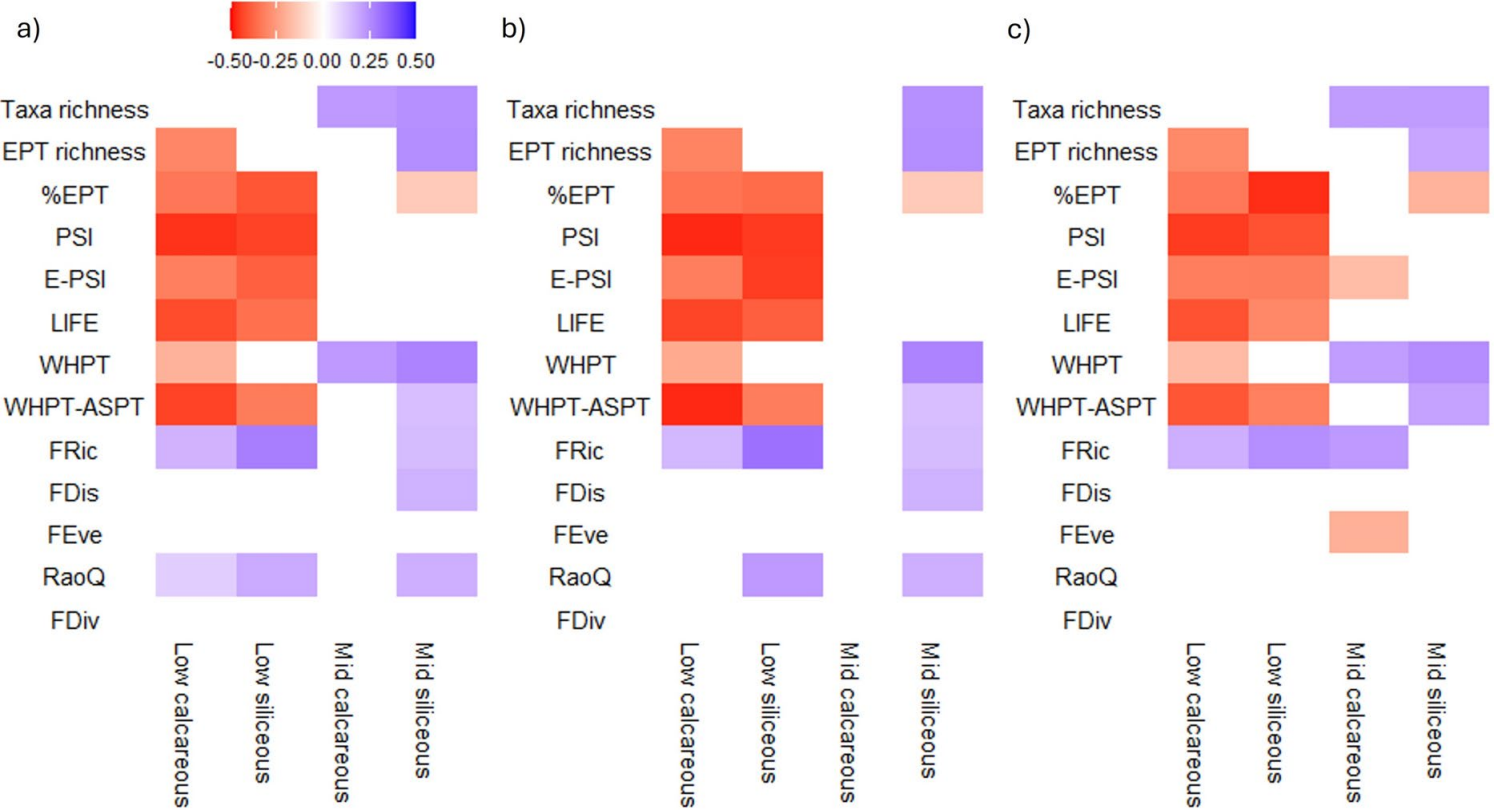
Correlation matrix for taxonomic and functional community measures with deposited fine sediment (% visual cover) for **a** spring and autumn combined, **b** spring and **c** autumn. Colour ramp indicates Spearman’s rank correlation coefficient. Only significant pairwise correlations (p < 0.05) are presented (Holm-Bonferroni corrected). For a full list of correlation coefficients (including insignificant associations) please see Table S2.

The associations of taxonomic and functional indices with deposited fine sediment were highly dependent on river typology (Fig. 4a). More indices showed significant correlations with deposited fine sediment in lowland rivers (specifically lowland calcareous) than in mid-altitude rivers. %EPT, PSI, E-PSI, LIFE, WHPT-Total and WHPT-ASPT all demonstrated significant negative correlations with increasing coverage of deposited fine sediment in both lowland river types (with EPT richness and WHPT-Total being significant for only lowland calcareous rivers). The single strongest pairwise association was with the sediment-specific metric PSI in lowland calcareous rivers. Taxa richness was only significantly associated with deposited fine sediment (positively) in mid altitude rivers. A number of metrics demonstrated contrasting directional responses between the two altitude categories. EPT richness displayed a moderate negative association in lowland calcareous, whilst a moderate positive association was recorded in mid-altitude siliceous rivers (Fig. 4a). Similarly, the water quality metrics of WHPT-Total and WHPT-ASPT demonstrated negative associations in lowland rivers, but positive associations in mid-altitude rivers. %EPT was the only metric to be significant and displayed the same directional response (negative) in both lowland and mid-altitude rivers (not mid-calcareous). Functional response metrics demonstrated a much weaker association with increasing coverage of deposited fine sediment. All statistically significant functional metrics displayed a positive association with deposited fine sediment, with FRic and RaoQ being the two most consistent metrics with responses in three of four river types, although these were all weak – moderate (Fig. 4a). Overall, lowland river types demonstrated stronger associations with response metrics, closely followed by mid-altitude siliceous, with mid-calcareous demonstrating few associations.

Considering sampling season, the sediment sensitive metric PSI demonstrated comparable strength associations in lowland river types in both seasons. E-PSI displayed a slight difference in the strength of association by season in addition to mid-altitude calcareous demonstrating a statistical association in autumn only (Fig. 4b, c). Overall, there were a comparable number of associations between ecological metrics and deposited fine sediment in spring and autumn with the majority being consistent between seasons. Notably, fine sediment in mid-altitude calcareous demonstrated no relationships with any metrics in spring (Fig. 4b, c). Of the functional metrics, FRic demonstrated the most consistent response across seasons with RaoQ only demonstrating weak associations in both silicious river types in spring (Fig. 4b, c). Full statistical outputs are available in Tables S2-S4.

## Discussion

Excess fine sediment is a pervasive stressor which can cause negative ecological consequences for aquatic ecosystems globally (Owens et al. 2005; McKenzie et al. 2024). However, our results demonstrate, for the first time, the varying responses of freshwater invertebrates to deposited fine sediment through the application of a river typology approach, with communities in some river types being more sensitive to deposited fine sediment than others. Whilst we cannot completely discount other factors influencing the thresholds identified, we observed that communities in mid-altitude rivers displayed a much lower threshold (~ < 10%) to deposited sediment, whilst those in lowland rivers were more tolerant, with change occurring further along the gradient (20–25%). This was true for both taxonomic and functional measures of the macroinvertebrate community. In the case of mid-altitude siliceous rivers, change occurred almost immediately for negatively responding taxa (i.e. those which decreased in relative abundance and/or occurrence in response to deposited fine sediment) potentially suggesting a higher degree of sensitivity for taxa within these rivers.

It should, however, be noted that within our study, the distribution of fine sediment is much reduced in mid-altitude rivers compared with lowland rivers where higher levels of fine sediment were frequently recorded (Figure A1). This is likely to influence the results obtained in our study, with higher community thresholds unable to be detected statistically. However, it is also highly likely that these reduced distributions in mid-altitude rivers also directly influence community sensitivity due to the communities being rarely exposed to fine sediment in excess quantities, unlike their lowland counterparts which are likely to have undergone environmental filtering (Matthaei et al. 2006; McKenzie et al. 2024). Two existing studies (Connolly and Pearson 2007; Mathers et al. 2022) have documented differing responses at the community level in experimental manipulations of sediment addition (artificial flumes and in-situ colonisation baskets respectively), with both reporting higher sensitivity for upland biological communities. Our results support and extend these observations to much larger strategic spatial scales (landscape) necessary for informing monitoring and management efforts.

Individual taxon responses across the fine sediment deposition gradient also varied by river typology and, as with community thresholds, the starkest differences occurred between the different altitude groupings. Of particular note was Heptageniidae, which appeared to be a key indicator taxa of deposited fine sediment pressure in all four river typologies, but which displayed varying change points. For mid-altitude rivers, peak change in their abundance occurred in general at < 10%; whilst for lowland calcareous rivers, this was at ca. 20% and for lowland siliceous their response threshold occurred at 50%. Gammaridae demonstrated an interesting indictor taxa response, representing a highly sensitive negative responder in mid-altitude siliceous rivers but responding positively in lowland silicious rivers. Species within both these families have been shown to display contrasting results to fine sediment coverage and therefore the taxonomic resolution of data may explain some of the contrasting change points (Gieswein et al. 2019). It is highly likely that species level identification could help identify nuanced differences in fine sediment community responses overlooked here, with this being particularly true of species that occur in different typologies (Extence et al. 2013).

*Gammarus* sp. are often used as model organisms in fine sediment studies, being moderately sensitive to fine sediment but able to burrow through some deposits (e.g. Vadher et al. 2015; Mathers et al. 2019). An experimental study by Conroy et al. (2018) exposed a range of species including one Heptageniidae representative, *Rhithrogena semicolorata,* and one Gammaridae, *Gammarus dubeni,* to fine sediment burial and reported that individuals from upland sources took longer to emerge from burial. It is therefore highly likely that the greater sensitivity of upland species is due to their naturally low exposure to deposited fine sediment, whilst lowland species are more frequently exposed to greater channel bed sediment loadings and thus may be less sensitive (Connolly and Pearson 2007). It is becoming more widely acknowledged that historic landscape exposure to excess fine sediment loading is a key factor in determining the present-day response of macroinvertebrate communities to fine sediment pressure (Matthaei et al. 2006; Larsen et al. 2009; Mathers et al. 2022; Mckenzie et al. 2024). Critically, our study herein represents the first landscape examination of these hypothesised differences in a categorical fashion to inform effective management strategies and monitoring.

The variability in community and taxon-specific fine sediment threshold responses between the river types raises important questions for how we monitor deposited fine sediment. The application of sediment-specific metrics is a rapid measure of riverine health and such metrics have been proven to demonstrate a strong relationship with fine sediment cover globally (Doretto et al. 2018; Mckenzie et al. 2022; Davis et al. 2024). Despite this, the effectiveness of these metrics may be influenced should communities / species demonstrate some plasticity / adaptation to increased deposited fine sediment (Connolly and Pearson 2007; Conroy et al. 2018), or have undergone historic environmental filtering due to lowland streams typically receiving long-term inputs of high amounts of fine sediment (Matthaei et al. 2006; McKenzie et al. 2024). We found that neither mid-altitude river types demonstrated an association with either of the sediment specific metrics (PSI or E-PSI). These sediment-specific indices are typically developed using a broad range of rivers and are currently applied indiscriminately in monitoring applications regardless of river type (Turley et al., 2014). %EPT, in contrast, did show a weak association in mid-siliceous rivers suggesting that perhaps these taxonomic groups are the best indicators of deposited fine sediment stress in terms of representing a ubiquitous tool across river typologies. This agrees with the findings of McKenzie et al. (2024) who found that EPT richness and %EPT represented the most consistent ecological tool across three continents constituting four countries (although note that no biomonitoring tools were tested). Other authors have observed that EPT metrics represent a relatively consistent and efficient tool in detecting fine sediment stress (Buendia et al. 2013; Doretto et al. 2018; McKenzie et al. 2022). However, it is also imperative that the ecological implications of enhanced levels of fine sediment deposition continue to be examined and monitored at both the community and individual taxon level as evidence is increasingly suggesting that biomonitoring indices and community metrics may respond to multiple stressors (Jones et al. 2023) or mask shifts in community composition with generalists replacing specialist taxa (Larsen et al. 2018; Mathers et al. 2024a, b).

Notably, functional response metrics demonstrated a much weaker association with deposited fine sediment. Previous studies have reported equivocal individual trait-sediment relationships reported in the literature (see Murphy et al. 2017; Wilkes et al. 2017 for summaries) which may reflect that stressors do not act on individual traits but many interacting traits which may make it hard to disentangle responses (Verberk et al. 2013). Further development of trait databases and indices may be required, or a new framework for linking functional responses to environmental pressures (Wilkes et al. 2020).

Lowland rivers, regardless of the geology, demonstrated the greatest association between community metrics and deposited fine sediment cover, with few associations in mid-altitude rivers. The implications of fine sediment coverage in mid-altitude rivers may be non-linear if these rivers are sediment supply-limited due to inputs being consistently low (dependent on surrounding land use and management), but also due to mid-altitude streams demonstrating sufficient stream power to transport fine material, unlike their lowland counterparts which are often transport limited (Naden et al. 2016). We observed that taxa richness, EPT richness, the water quality metric of WHPT and a number of functional metrics demonstrated a positive association with increasing deposited fine sediment cover in mid-altitude rivers. Indeed, TITAN analysis suggested wide confidence intervals for taxa responding positively to deposited fine sediment in mid-altitude rivers with the peak occurring at low coverage (~ < 5%) but the 95th percentile extending to around 35% coverage. In addition, the number of reliable indicator taxa from TITAN was overwhelmingly dominated by positively responding taxa in mid-altitude river types. Clearly some level of fine sediment delivery into these rivers provides a beneficial resource/habitat for some species (Carlson et al. 2013). Wagenhoff et al. (2011) speculated that fine sediment may act as a subsidy in low quantities but found no evidence to support this hypothesis. However, this may be because although they sampled extensively over the fine sediment gradient, no account was made for the confounding effects of typology / land use/stream power.

Fine sediment, particularly when delineated further into sand / silt sediment fractions (proxy for inorganic/organic), likely demonstrates strong resource implications for many lotic ecosystems, particularly when assessed seasonally. Mathers et al., (2023; 2024a, b) demonstrated that, in many river systems, the invertebrate richness supported by sand and silt is no different to that provided by gravel, and that silt patches demonstrate strong links associated with seasonally variable resource availability/utilisation. Our study found that, in general, negatively responding community change points were fairly consistent between seasons, whilst positively responding taxa were more seasonally dependent. A number of taxa occurred as reliable indicators in one season but not in the other and the location of change points along the gradient differed for some taxa (e.g., Caenidae and Chloroperlidae). Our results suggest that sampling season may have family-specific implications for evaluating the effects of fine sediment deposition but that communities as a whole are less temporally sensitive, agreeing with a number of other field studies (McKenzie et al. 2022; Davis et al. 2024). However, Mathers et al. (2017) recorded that fine sediment loading implications for community structure were not temporally consistent, most likely associated with taxon life-histories, but their study was conducted at fine resolution (14-day periods over 126 days) and undertook experimental manipulation of the fine sediment loading.

Seasonal variation is widely known to affect ecological assessments (Sporka et al., 2006; Clarke and Hering 2006; Johnson et al. 2012). We observed that, in general, the direction of association of community response metrics with deposited fine sediment remained consistent regardless of the season. However, the strength of these associations varied seasonally. In addition, mid-altitude calcareous rivers only demonstrated metric associations in autumn and not spring. Notably, the sediment sensitive PSI metric demonstrated associations of comparable strength in lowland river types in both seasons. Further research is required to disentangle the timing of sediment inputs and exfiltration as little work has considered this aspect. We currently have a poor understanding of the appropriate sampling frequency required to capture deposited fine sediment levels and the implications for invertebrate communities (Davis et al. 2024).

## Conclusion

It is clear that deposited fine sediment does not produce linear responses (McKenzie et al. 2024) as has been observed for other gradients such as glacial cover and urbanisation (Brown et al. 2018; Chen and Olden 2020). Here, we provide evidence to suggest that, even within geographical regions, the response to deposited fine sediment is likely to be non-linear, associated with a number of environmental controls on the deposition, accumulation and storage of fine sediment (Naden et al. 2016) and potentially confounded by the timing of inputs/sample season (Carlson et al. 2013). However, our study demonstrates that the application of the river typology classification to assess the ecological implications of deposited fine sediment may be a useful tool to form the foundation of future research, management and monitoring efforts, with differential invertebrate responses and change points identified based on the river type. We urge researchers and environmental regulators to consider carefully how deposited fine sediment is assessed and how its role is valued in lotic ecosystems. We advocate that future research considers the context-specific effects of fine sedimentation with our study suggesting that deposited fine sediment may not act as a stressor at very low levels in all river systems, particularly those that are located upstream in the catchment where it may act as a resource.

## Supplementary Information

Below is the link to the electronic supplementary material.

Supplementary file1 (DOCX 2168 kb)

Supplementary file2 (PPTX 11549 kb)

## Data Availability

The Environment Agency data used in this study are freely available from https:// environment.data.gov.uk/ecology/explorer/. Other data employed in the study are available from the corresponding author on reasonable request.
