# Supplementary material for: Context matters: how river typology shapes biotic responses to fine sediment pressure
Source: Landsc Ecol. 2026 Jan 28;41(2):41. doi: 10.1007/s10980-026-02297-z (PMC12890971; doi:10.1007/s10980-026-02297-z)
Supplement: Supplementary file 1 — Supplementary file1 (DOCX 2168 kb) [file 10980_2026_2297_MOESM1_ESM.docx]

**Data sources used in study**

The Environment Agency, the regulatory authority in England, routinely collects invertebrate data for monitoring purposes and biological data is openly available online via the Ecology and Fish Data Explorer (<https://environment.data.gov.uk/ecology/explorer/>). Sample sites were screened to retain those that fulfilled the following criteria: (a) upstream catchment area greater than 10 km^2^, (b) was not downstream of discharge from a sewage treatment plant, (c) diffuse sources at associated water quality monitoring site accounted for at least 70% of the total pollutant load on sampled days; (d) physicochemical parameters (pH, temperature, ammonia, phosphate and dissolved oxygen) were not classified as ‘Poor’ or ‘Bad’ according to Water Framework Directive (WFD) (2000/60/EC) classification; and (e) ecological parameters (invertebrates) were not classified as ‘Moderate’, ‘Poor’ or ‘Bad’.

Only data collected in either spring (March - May) or autumn (September - November) were retained. The Environment Agency follows standard protocols for sampling, processing, and identification of invertebrate samples. Samples were collected via the standard multi-habitat 3-minute kick survey (1 mm mesh size) followed by a 1-minute hand search for surface dwelling or animals attached to logs, stones, overhanging vegetation or other solid objects (Environment Agency, 2014a). Sampling time was distributed proportionally between the habitats present in the sampling area (e.g., riffle, marginal, pool etc). Following collection, the contents of the pond net were preserved (100% Industrial Methylated Spirits) and samples processed following Environment Agency operational instructions for the sorting and identification of invertebrates in the laboratory (Environment Agency, 2014b). Invertebrates were identified to mixed taxon level with most insect taxa identified to species level. Visual estimates of the substrate composition were carried out using Wentworth categories: boulders (>256 mm), cobbles (64 – 256 mm), pebbles (16 – 64 mm), gravel (2 – 16 mm), sand (0.06 – 2 mm), silt (<0.06 mm), and clay (<0.06 mm sticky and cohesive).

A second data source collected for academic research (Murphy et al., 2015, 2017) was also collated that included additional sites from rural catchments in England and Wales. Sites were selected that were subject to fine sediment stress predominantly from agricultural sources (>75% as a proportion of modelled total sediment input (Strömqvist et al., 2008)) and were not affected by confounding disturbance pressures (e.g., downstream of sewage treatment works, lakes and reservoirs, or urban areas). The data source included sites from a range of river types and levels of sediment pressures. Additional data from headwater streams in Wales (Jones et al., 2017) were provided from sites located in independent watersheds in agricultural catchments with low urban/suburban (<10%) and forestry (<10%) cover. All the Welsh sites were sampled in spring and autumn. As with the Environment Agency data, invertebrates were sampled using a standard 3-minute kick (1 mm mesh size) followed by a 1-minute hand search. Habitats were sampled proportional to their total coverage within the reach. Invertebrates were fixed (10% formalin) and identified in a laboratory to mixed taxon level.


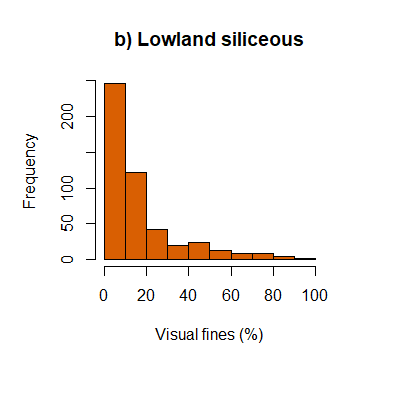

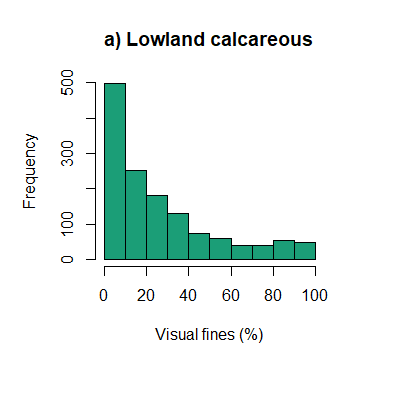

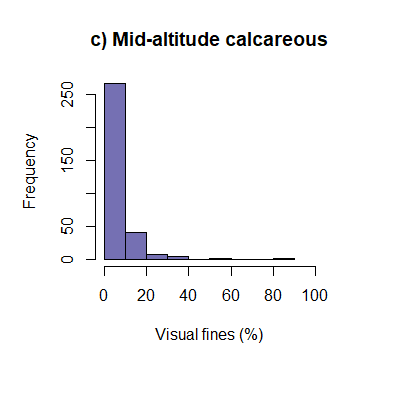

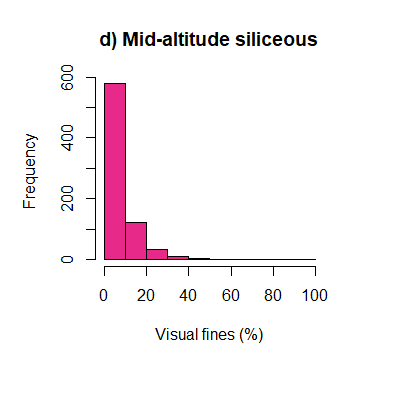


**Figure A1.** Histograms of fine sediment gradient (as visual estimates %) for each river typology.

**References**

Environment Agency. (2014a). *Freshwater macro-invertebrate sampling in rivers. Operational instruction 018_08.*

Environment Agency. (2014b). *Freshwater macro-invertebrate analysis of riverine samples. Operational instruction 024_08*.

Murphy, J. F., Jones, J. I., Arnold, A., Duerdoth, C. P., Pretty, J. L., Naden, P. S., Sear, D. A., & Collins, A. L. (2017). Can macroinvertebrate biological traits indicate fine-grained sediment conditions in streams? *River Research and Applications*, *33*(10), 1606–1617. https://doi.org/10.1002/rra.3194

Murphy, J. F., Jones, J. I., Pretty, J. L., Duerdoth, C. P., Hawczak, A., Arnold, A., Blackburn, J. H., Naden, P. S., Old, G., Sear, D. A., Hornby, D., Clarke, R. T., & Collins, A. L. (2015). Development of a biotic index using stream macroinvertebrates to assess stress from deposited fine sediment. *Freshwater Biology*, *60*(10), 2019–2036. https://doi.org/10.1111/fwb.12627

Strömqvist, J., Collins, A. L., Davison, P. S., & Lord, E. I. (2008). PSYCHIC – A process-based model of phosphorus and sediment transfers within agricultural catchments. Part 2. A preliminary evaluation. *Journal of Hydrology*, *350*(3–4), 303–316.

Jones, J. I., Murphy, J. F., Anthony, S. G., Arnold, A., Blackburn, J. H., Duerdoth, C. P., Hawczak, A., Hughes, G. O., Pretty, J. L., Scarlett, P. M., Gooday, R. D., Zhang, Y. S., Fawcett, L. E., Simpson, D., Turner, A. W. B., Naden, P. S., & Skates, J. (2017). Do agri-environment schemes result in improved water quality? *Journal of Applied Ecology*, *54*(2), 537–546. <https://doi.org/10.1111/1365-2664.12780>.

McKenzie, M., Brooks, A., Callisto, M., Collins, A.L., Durkota, J.M., Death, R.G., Jones, J.I., Linares, M.S., Matthaei, C.D., Monk, W.A. and Murphy, J.F., 2024. Freshwater invertebrate responses to fine sediment stress: A multi‐continent perspective. *Global Change Biology*, *30*(1), p.e17084.
