# Supplementary material for: Context matters: how river typology shapes biotic responses to fine sediment pressure
Source: Landsc Ecol. 2026 Jan 28;41(2):41. doi: 10.1007/s10980-026-02297-z (PMC12890971; doi:10.1007/s10980-026-02297-z)
Supplement: Supplementary file 2 — Supplementary file2 (PPTX 11549 kb) [file 10980_2026_2297_MOESM2_ESM.pptx]

## Slide 1
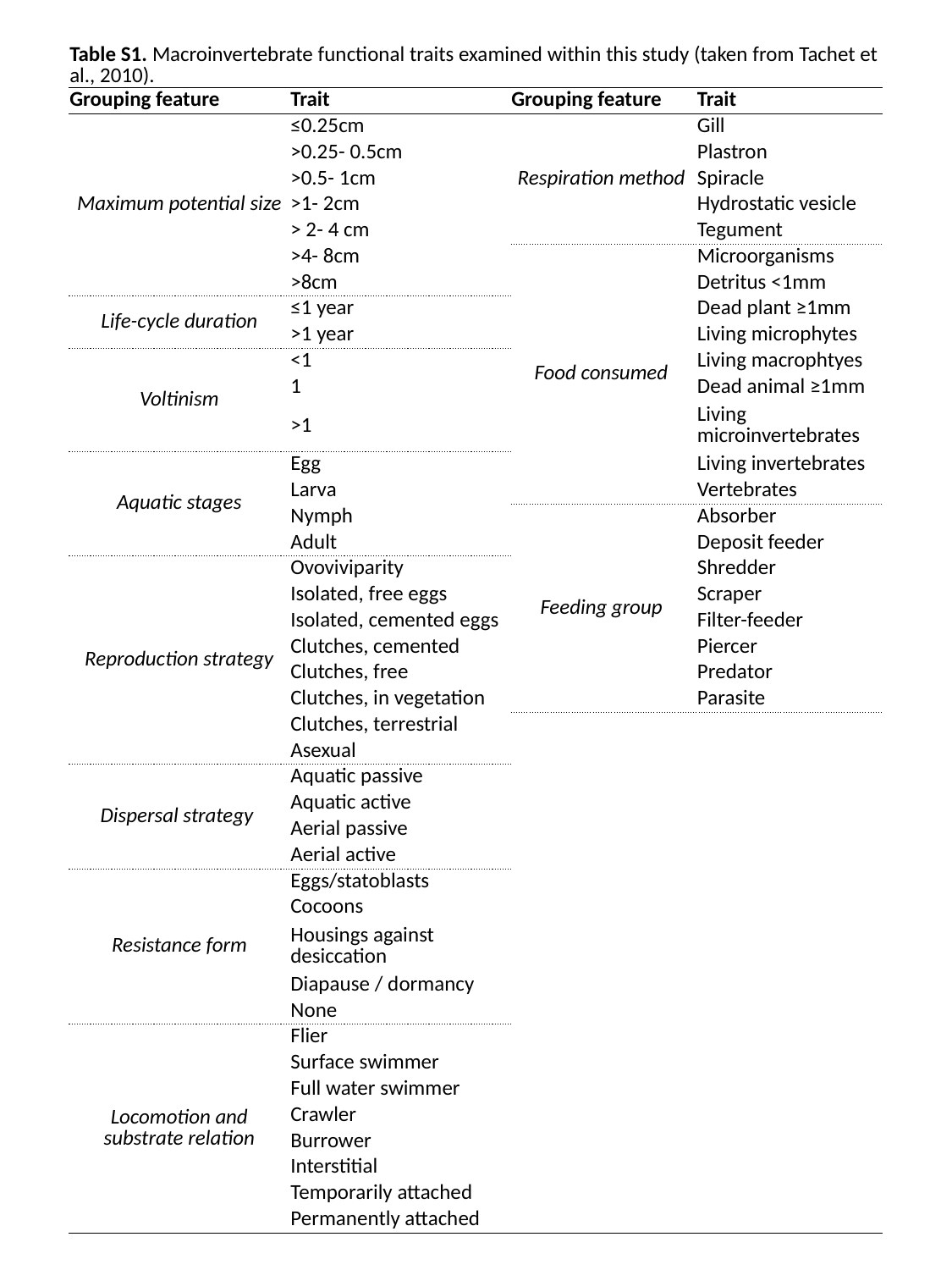

| Table S1. Macroinvertebrate functional traits examined within this study (taken from Tachet et al., 2010). | | | |
| --- | --- | --- | --- |
| Grouping feature | Trait | Grouping feature | Trait |
| Maximum potential size | ≤0.25cm | Respiration method | Gill |
| | >0.25- 0.5cm | | Plastron |
| | >0.5- 1cm | | Spiracle |
| | >1- 2cm | | Hydrostatic vesicle |
| | > 2- 4 cm | | Tegument |
| | >4- 8cm | Food consumed | Microorganisms |
| | >8cm | | Detritus <1mm |
| Life-cycle duration | ≤1 year | | Dead plant ≥1mm |
| | >1 year | | Living microphytes |
| Voltinism | <1 | | Living macrophtyes |
| | 1 | | Dead animal ≥1mm |
| | >1 | | Living microinvertebrates |
| Aquatic stages | Egg | | Living invertebrates |
| | Larva | | Vertebrates |
| | Nymph | Feeding group | Absorber |
| | Adult | | Deposit feeder |
| Reproduction strategy | Ovoviviparity | | Shredder |
| | Isolated, free eggs | | Scraper |
| | Isolated, cemented eggs | | Filter-feeder |
| | Clutches, cemented | | Piercer |
| | Clutches, free | | Predator |
| | Clutches, in vegetation | | Parasite |
| | Clutches, terrestrial | | |
| | Asexual | | |
| Dispersal strategy | Aquatic passive | | |
| | Aquatic active | | |
| | Aerial passive | | |
| | Aerial active | | |
| Resistance form | Eggs/statoblasts | | |
| | Cocoons | | |
| | Housings against desiccation | | |
| | Diapause / dormancy | | |
| | None | | |
| Locomotion and substrate relation | Flier | | |
| | Surface swimmer | | |
| | Full water swimmer | | |
| | Crawler | | |
| | Burrower | | |
| | Interstitial | | |
| | Temporarily attached | | |
| | Permanently attached | | |

## Slide 2
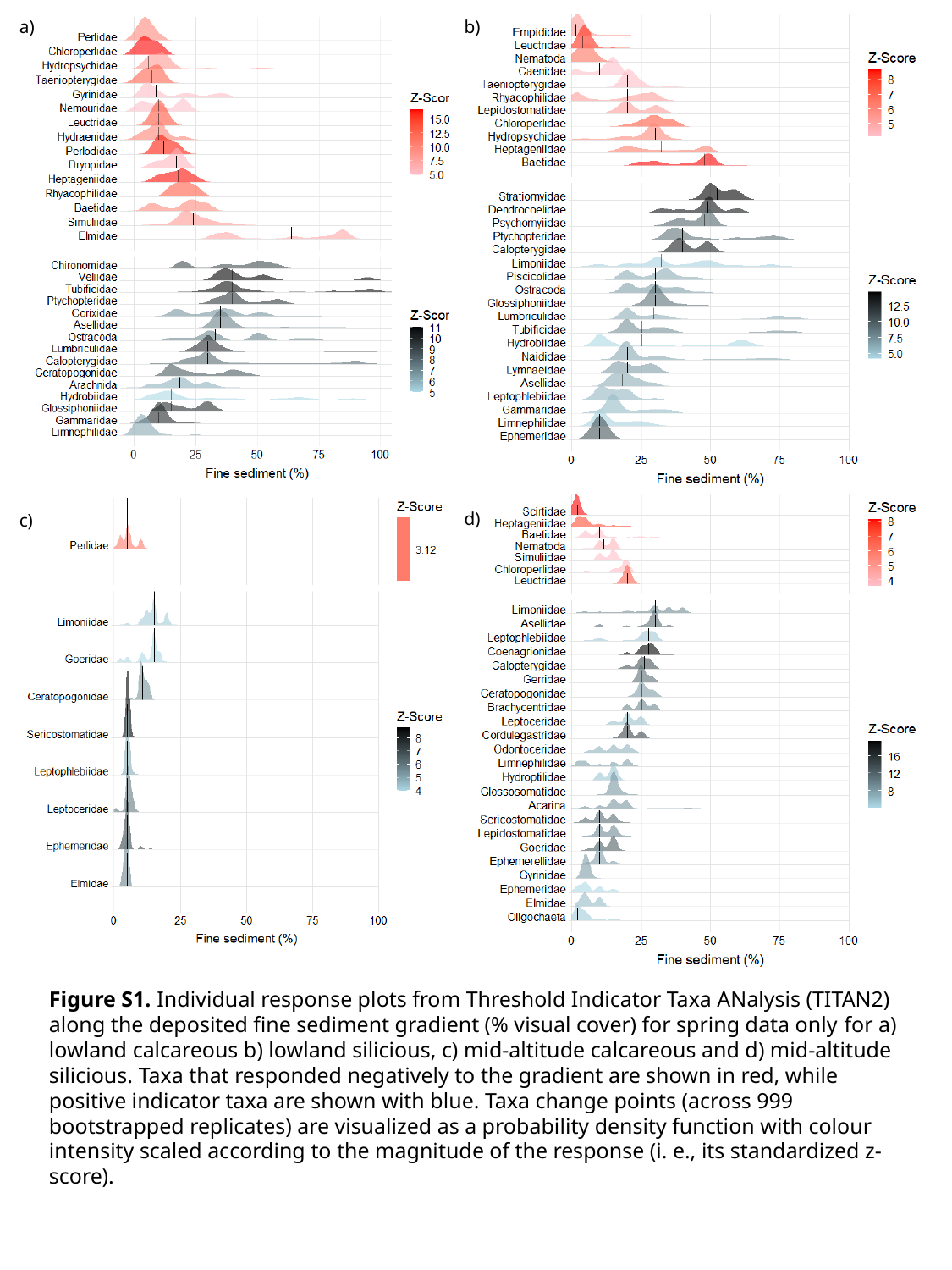

a)
b)
d)
c)
Figure S1. Individual response plots from Threshold Indicator Taxa ANalysis (TITAN2) along the deposited fine sediment gradient (% visual cover) for spring data only for a) lowland calcareous b) lowland silicious, c) mid-altitude calcareous and d) mid-altitude silicious. Taxa that responded negatively to the gradient are shown in red, while positive indicator taxa are shown with blue. Taxa change points (across 999 bootstrapped replicates) are visualized as a probability density function with colour intensity scaled according to the magnitude of the response (i. e., its standardized z-score).

## Slide 3
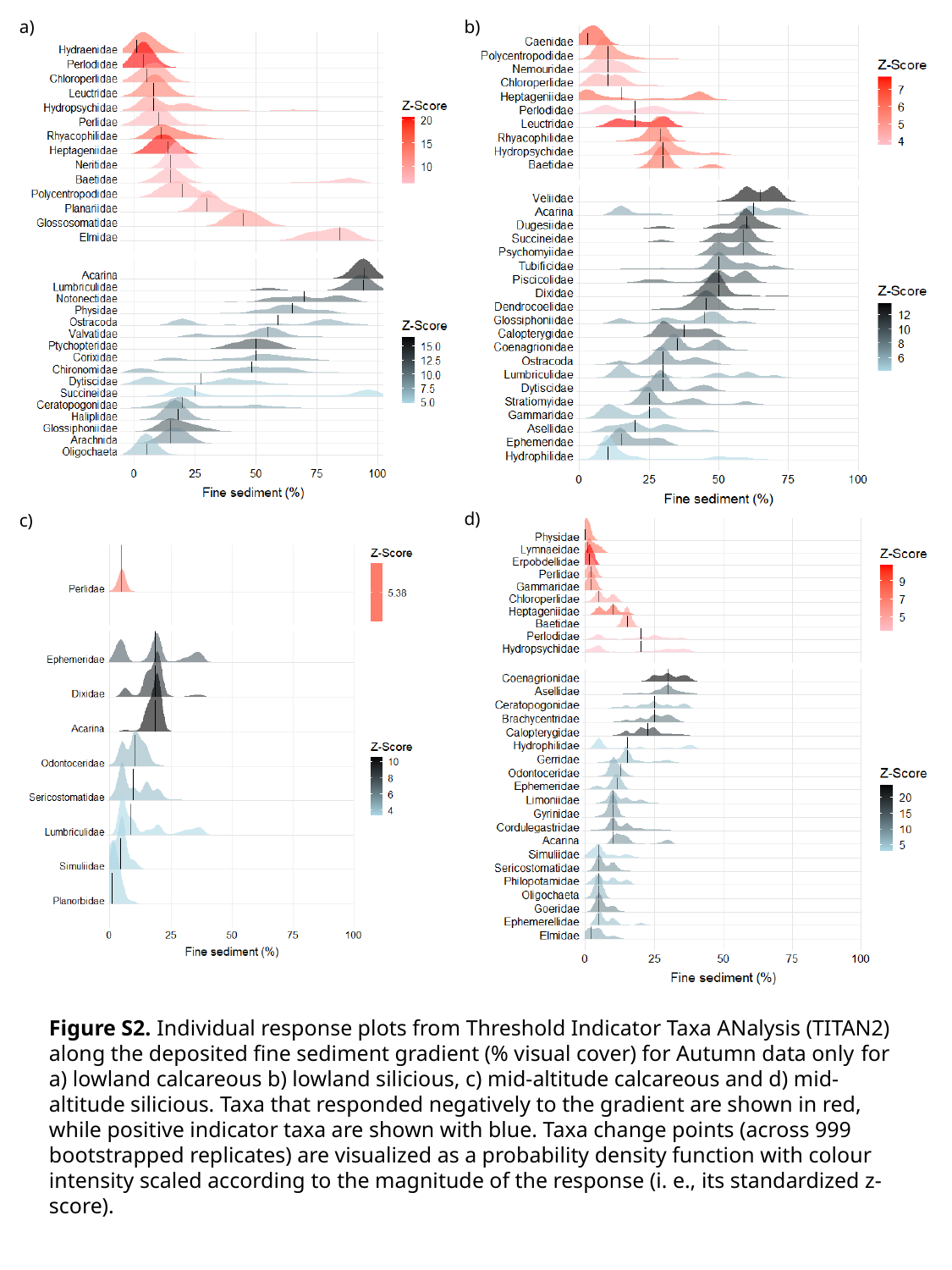

a)
b)
d)
c)
Figure S2. Individual response plots from Threshold Indicator Taxa ANalysis (TITAN2) along the deposited fine sediment gradient (% visual cover) for Autumn data only for a) lowland calcareous b) lowland silicious, c) mid-altitude calcareous and d) mid-altitude silicious. Taxa that responded negatively to the gradient are shown in red, while positive indicator taxa are shown with blue. Taxa change points (across 999 bootstrapped replicates) are visualized as a probability density function with colour intensity scaled according to the magnitude of the response (i. e., its standardized z-score).

## Slide 4
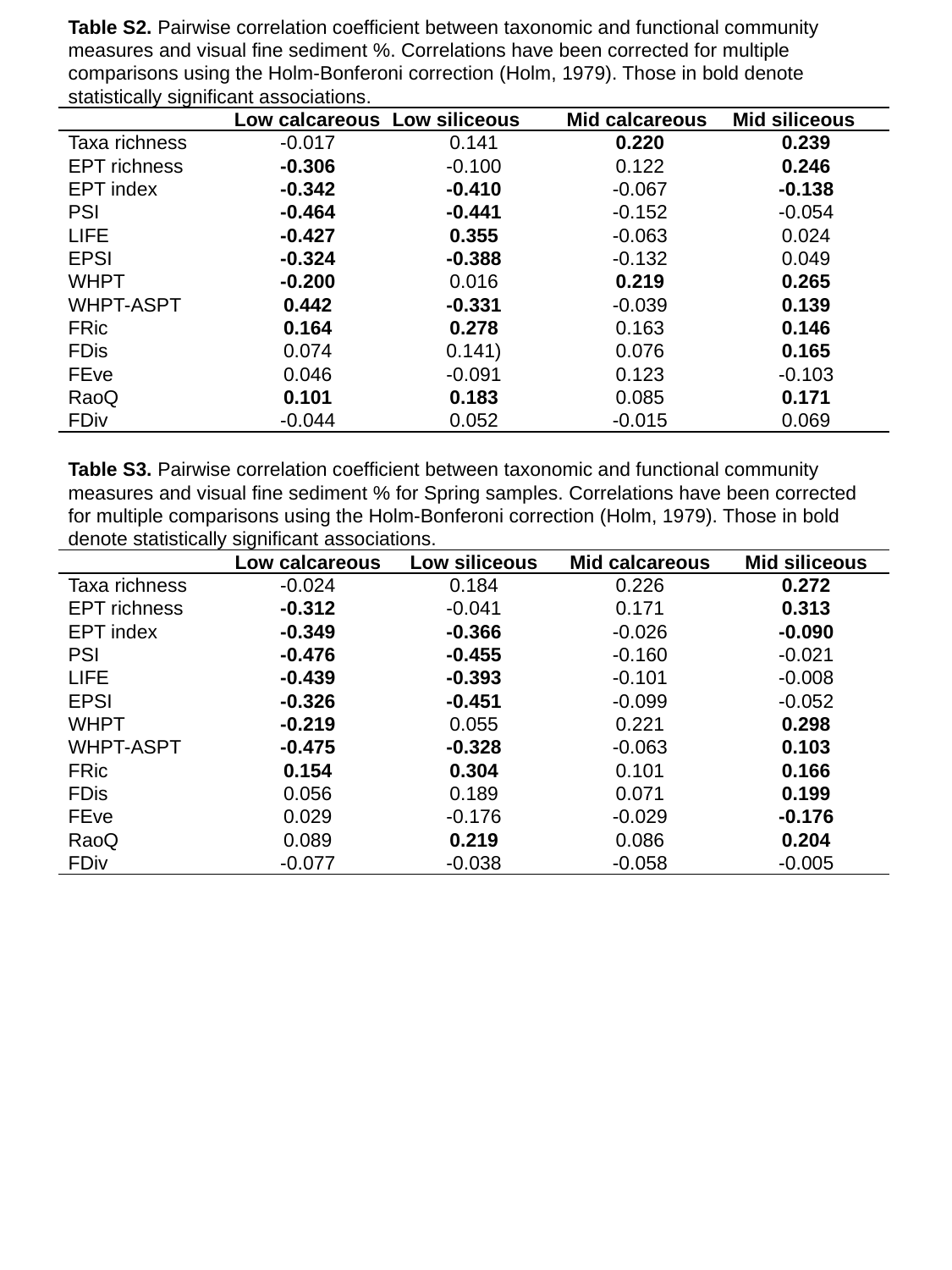

| Table S2. Pairwise correlation coefficient between taxonomic and functional community measures and visual fine sediment %. Correlations have been corrected for multiple comparisons using the Holm-Bonferoni correction (Holm, 1979). Those in bold denote statistically significant associations. | | | | |
| --- | --- | --- | --- | --- |
| | Low calcareous | Low siliceous | Mid calcareous | Mid siliceous |
| Taxa richness | -0.017 | 0.141 | 0.220 | 0.239 |
| EPT richness | -0.306 | -0.100 | 0.122 | 0.246 |
| EPT index | -0.342 | -0.410 | -0.067 | -0.138 |
| PSI | -0.464 | -0.441 | -0.152 | -0.054 |
| LIFE | -0.427 | 0.355 | -0.063 | 0.024 |
| EPSI | -0.324 | -0.388 | -0.132 | 0.049 |
| WHPT | -0.200 | 0.016 | 0.219 | 0.265 |
| WHPT-ASPT | 0.442 | -0.331 | -0.039 | 0.139 |
| FRic | 0.164 | 0.278 | 0.163 | 0.146 |
| FDis | 0.074 | 0.141) | 0.076 | 0.165 |
| FEve | 0.046 | -0.091 | 0.123 | -0.103 |
| RaoQ | 0.101 | 0.183 | 0.085 | 0.171 |
| FDiv | -0.044 | 0.052 | -0.015 | 0.069 |
| Table S3. Pairwise correlation coefficient between taxonomic and functional community measures and visual fine sediment % for Spring samples. Correlations have been corrected for multiple comparisons using the Holm-Bonferoni correction (Holm, 1979). Those in bold denote statistically significant associations. | | | | |
| --- | --- | --- | --- | --- |
| | Low calcareous | Low siliceous | Mid calcareous | Mid siliceous |
| Taxa richness | -0.024 | 0.184 | 0.226 | 0.272 |
| EPT richness | -0.312 | -0.041 | 0.171 | 0.313 |
| EPT index | -0.349 | -0.366 | -0.026 | -0.090 |
| PSI | -0.476 | -0.455 | -0.160 | -0.021 |
| LIFE | -0.439 | -0.393 | -0.101 | -0.008 |
| EPSI | -0.326 | -0.451 | -0.099 | -0.052 |
| WHPT | -0.219 | 0.055 | 0.221 | 0.298 |
| WHPT-ASPT | -0.475 | -0.328 | -0.063 | 0.103 |
| FRic | 0.154 | 0.304 | 0.101 | 0.166 |
| FDis | 0.056 | 0.189 | 0.071 | 0.199 |
| FEve | 0.029 | -0.176 | -0.029 | -0.176 |
| RaoQ | 0.089 | 0.219 | 0.086 | 0.204 |
| FDiv | -0.077 | -0.038 | -0.058 | -0.005 |

## Slide 5
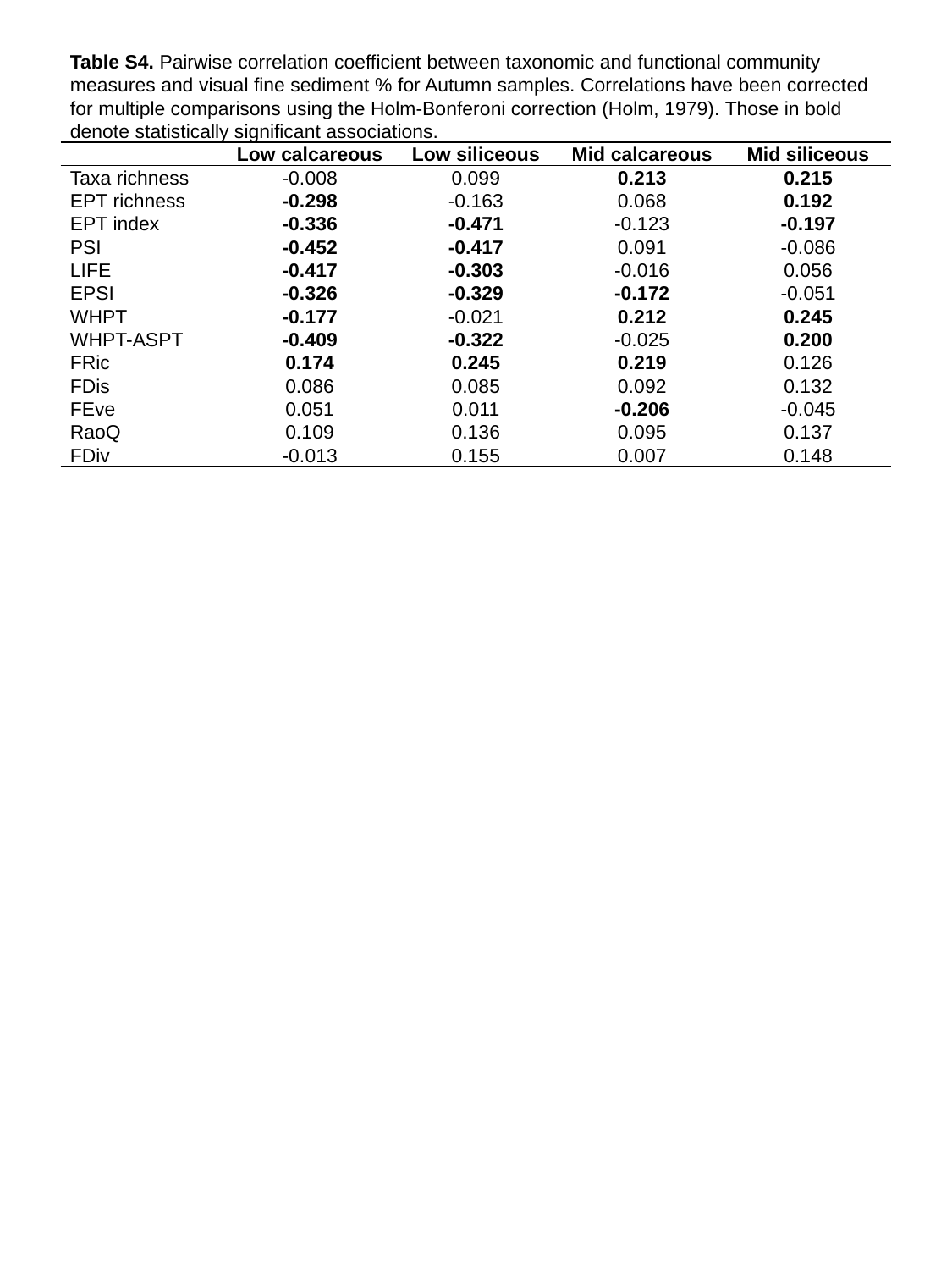

| Table S4. Pairwise correlation coefficient between taxonomic and functional community measures and visual fine sediment % for Autumn samples. Correlations have been corrected for multiple comparisons using the Holm-Bonferoni correction (Holm, 1979). Those in bold denote statistically significant associations. | | | | |
| --- | --- | --- | --- | --- |
| | Low calcareous | Low siliceous | Mid calcareous | Mid siliceous |
| Taxa richness | -0.008 | 0.099 | 0.213 | 0.215 |
| EPT richness | -0.298 | -0.163 | 0.068 | 0.192 |
| EPT index | -0.336 | -0.471 | -0.123 | -0.197 |
| PSI | -0.452 | -0.417 | 0.091 | -0.086 |
| LIFE | -0.417 | -0.303 | -0.016 | 0.056 |
| EPSI | -0.326 | -0.329 | -0.172 | -0.051 |
| WHPT | -0.177 | -0.021 | 0.212 | 0.245 |
| WHPT-ASPT | -0.409 | -0.322 | -0.025 | 0.200 |
| FRic | 0.174 | 0.245 | 0.219 | 0.126 |
| FDis | 0.086 | 0.085 | 0.092 | 0.132 |
| FEve | 0.051 | 0.011 | -0.206 | -0.045 |
| RaoQ | 0.109 | 0.136 | 0.095 | 0.137 |
| FDiv | -0.013 | 0.155 | 0.007 | 0.148 |
